# Neural traces of stress: cortisol related sustained enhancement of amygdala-hippocampal functional connectivity

**DOI:** 10.3389/fnhum.2013.00313

**Published:** 2013-07-05

**Authors:** Sharon Vaisvaser, Tamar Lin, Roee Admon, Ilana Podlipsky, Yona Greenman, Naftali Stern, Eyal Fruchter, Ilan Wald, Daniel S. Pine, Ricardo Tarrasch, Yair Bar-Haim, Talma Hendler

**Affiliations:** ^1^Functional Brain Center, Wohl Institute for Advanced Imaging, Sourasky Medical CenterTel-Aviv, Israel; ^2^Sackler Faculty of Medicine, Tel Aviv UniversityTel Aviv, Israel; ^3^Sagol School of Neuroscience, Tel Aviv UniversityTel Aviv, Israel; ^4^School of Psychological Sciences, Tel Aviv UniversityTel Aviv, Israel; ^5^Center for Depression, Anxiety, and Stress Research, Harvard Medical School, McLean HospitalBelmont, MA, USA; ^6^Sourasky Medical Center, Institute of Endocrinology, Metabolism, and HypertensionTel Aviv, Israel; ^7^Division of Mental Health, Medical Corps, IDFTel Hashomer, Israel; ^8^Section on Development and Affective Neuroscience of the National Institute of Mental Health, National Institutes of HealthBethesda, MD, USA; ^9^School of Education, Tel Aviv UniversityTel Aviv, Israel

**Keywords:** fMRI, resting-state functional connectivity, default-mode network, recovery, limbic connectivity

## Abstract

Stressful experiences modulate neuro-circuitry function, and the temporal trajectory of these alterations, elapsing from early disturbances to late recovery, heavily influences resilience and vulnerability to stress. Such effects of stress may depend on processes that are engaged during resting-state, through active recollection of past experiences and anticipation of future events, all known to involve the default mode network (DMN). By inducing social stress and acquiring resting-state functional magnetic resonance imaging (fMRI) before stress, immediately following it, and 2 h later, we expanded the time-window for examining the trajectory of the stress response. Throughout the study repeated cortisol samplings and self-reports of stress levels were obtained from 51 healthy young males. Post-stress alterations were investigated by whole brain resting-state functional connectivity (rsFC) of two central hubs of the DMN: the posterior cingulate cortex (PCC) and hippocampus. Results indicate a ’recovery’ pattern of DMN connectivity, in which all alterations, ascribed to the intervening stress, returned to pre-stress levels. The only exception to this pattern was a stress-induced rise in amygdala-hippocampal connectivity, which was sustained for as long as 2 h following stress induction. Furthermore, this sustained enhancement of limbic connectivity was inversely correlated to individual stress-induced cortisol responsiveness (AUCi) and characterized only the group lacking such increased cortisol (i.e., *non-responders*). Our observations provide evidence of a prolonged post-stress response profile, characterized by both the comprehensive balance of most DMN functional connections and the distinct time and cortisol dependent ascent of intra-limbic connectivity. These novel insights into neuro-endocrine relations are another milestone in the ongoing search for individual markers in stress-related psychopathologies.

## Introduction

Psychological stress is prevalent and strongly related to mental illnesses. The brain mediates stress responses and thus influences the individual's capacity to cope with them. Such coping depends on functions manifesting during several stages of the stress response, evolving gradually from early disturbances to later recovery and homeostasis restitution; processes that ultimately support the link between stress and psychopathology (McEwen, 2004; Yehuda and LeDoux, 2007). These processes may involve various internally-driven mental processes, such as drawing on past experiences and envisioning future events, known to increase during resting-state (Gruberger et al., 2011) and are therefore expected to be mediated by the default mode network (DMN) (Greicius et al., 2003; Buckner and Carroll, 2007). The DMN, defined as a cluster of regions deactivated during task performance and activated at rest, includes mainly the posterior cingulate cortex (PCC), medial prefrontal cortex (mPFC), inferior parietal lobule (IPL) and the hippocampal formation (Buckner et al., 2008). The relevance of integrated DMN activation during rest to stress-related psychopathology has been demonstrated by abnormal DMN connectivity in post-traumatic stress disorder (Bluhm et al., 2009; Rabinak et al., 2011) and depression (Greicius et al., 2007). Monitoring changes in resting-state functional connectivity (rsFC) was established as a tool for the identification of whole brain spontaneous co-activation clustering in functional magnetic resonance imaging (fMRI) (Fox and Raichle, 2007). Previous research highlights the importance of rest to mental homeostasis by demonstrating that cognitive and affective tasks have prolonged affects on neural activity at rest (Waites et al., 2005; Pyka et al., 2009; Eryilmaz et al., 2011). Therefore, it is reasonable to expect that stress will also show similar traces. Indeed, recent studies demonstrated modified amygdala rsFC, up to 1 h following stress (Van Marle et al., 2010; Veer et al., 2011). Despite preliminary evidence for the effect of stress on rsFC, the chronometry of such effects remains relatively limited and poorly specified.

The present study examined the chronometry of stress-rsFC relationships using three “rest” conditions: before stress induction, immediately after, and following a 90 min recess outside the scanner. Stress elicitation was achieved using a documented procedure for the induction of social stress via arithmetic calculations, monitored on-line (Wang et al., 2005, 2007; Gray et al., 2007). We selected two core DMN hubs as seeds for rsFC analyses; the PCC and hippocampus (Greicius et al., 2004; Buckner et al., 2008). The PCC has been documented as a pivotal node of the DMN that directly interacts with all other network nodes (Fransson and Marrelec, 2008; De Pasquale et al., 2012). Furthermore, both the hippocampus and PCC have been previously shown to be involved in stress responsiveness (Pruessner et al., 2008). Finally, we repeatedly measured subjective stress intensity, heart rate (HR), and cortisol levels.

We anticipated a decline in the stress response by the third rest condition, 2 h post stressor-task, generating rsFC patterns similar to those observed at baseline. We further hypothesized that recovery dynamics for rsFC with the hippocampus, an area supporting affective memory of the stressful experiences, would differ from the dynamics in other regions, and be individually determined by stress indices (e.g., cortisol response, stress rating).

## Materials and methods

### Participants

The study was conducted on 61 healthy males (age 19–22). Participants consisted of mandatory military soldiers who volunteered to participate in our study. All participants were positioned in the same service unit, amidst the same military course, and before operational employment. Of the 61 individuals 4 were excluded from cortisol analysis due to an insufficient saliva samples and 10 were excluded from the fMRI data analysis due to signal artifacts. Participants had no reported history of psychiatric or neurological disorders, no current use of psychoactive drugs, no family history of major psychiatric disorders, and no previous exposure to abuse during childhood and/or potentially traumatic events before entering the study. In addition, all participants had normal or corrected-to-normal vision and provided written informed consent approved by Tel Aviv Sourasky Medical Center Ethics Committee and conformed to the Code of Ethics of the World Medical Association (Helsinki Declaration).

### Experimental procedure

The experiment was performed at the Wohl Institute for Advanced Imaging in Tel Aviv. To minimize unwanted effects on cortisol levels, participants were awake for at least 3 h before arriving at the institute, and were instructed to eat breakfast and avoid further food intake, nicotine, caffeine, and exercise for at least 2 h before arrival. The study consisted of four phases: acclimation (15 min), 1st session in the MRI scanner (65 min), intermission (90 min), and a 2nd session in the MRI scanner (30 min). In the acclimation phase, participants were given a 15-min resting period, signed the informed consent forms and were introduced to the experimental procedure. In the 1st session in the MRI scanner participants underwent the acute stress task. Three “rest” conditions were integrated into the study design: before the tasks (“rest 1”), immediately afterwards (“rest 2”), and at the beginning of the 2nd scanning session, following a 90 min recess outside the scanner (“rest 3”). During the intermission phase outside the scanner participants completed questionnaires and were given a light meal. In “rest” conditions (5 min each) participants were instructed to stare at a fixation point in the center of a screen.

### Stress elicitation task

**Figure 1:**
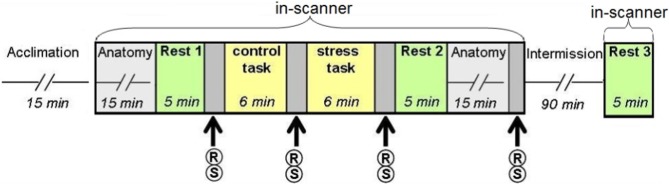
**Study design.** Following the acclimation phase, participants underwent two scanning sessions: the first included two “rest” conditions interspersed with the control (backward counting) and stress (serial subtraction) tasks; the second session, following a 90 min intermission outside the scanner, included a third “rest” scan. R, report of stress level; S, salivary cortisol sampling.

Acute stress was induced via a serial subtraction arithmetic task (Wang et al., 2005), a component of the Trier Social Stress Test (Kirschbaum et al., 1993) incorporated into the scanner. Participants were instructed to continuously subtract 13 from 1022 for a period of 6 min, and respond verbally, while the experimenters monitored and communicated with each subject on-line, constantly demanding faster and more accurate performance. A timer appeared at the top left corner of the screen to indicate to the participant how much time had elapsed. The stress task was preceded by a non-stressful condition-backward counting from 1000 for a period of 6 min, without external monitoring (Figure 1).

### Physiological and behavioral data collection

Psychological and physiological effects of stress were evaluated at 4 time points (Figure 1) through repeated self reports of stress levels (on a 9 point Likert scale, marked as R) and salivary cortisol sampling (marked as S), collected with a cotton swab, placed in the participants mouth for 3 min (Sarstedt, Numbrecht, Germany). Samples were stored at −20°C immediately after collection until further analyzed. Salivary concentrations of cortisol rose to peak levels 15–30 min after stress (De Kloet et al., 2005). Due to delayed peripheral response, final cortisol samples were obtained 20 min post-stress. Following peak measurements, cortisol levels gradually decline to pre-stress levels 60–90 min later (De Kloet et al., 2005). To avoid leakage of the effect, participants did not perform any additional tasks during this time interval.

### Endocrine data analysis

Salivary cortisol levels were assayed using Coat-A-Count radioimmunoassay (Siemens, Los Angeles, CA), inter- and intra-assay coefficient of variation (CV) 14.4%, 8.9%, respectively. Inter-assay % CVs of less than 15 and intra-assay % CVs of less than 10 are considered to indicate assays with good and reliable performance. In order to obtain a reliable measure of the individual's cortisol reactivity, in accordance to the expected gradual ascent, we calculated the area under the curve increase (AUCi) using the equation from Pruessner et al. (2003), with trapezoidal integration.

### Electrophysiological data collection and analysis

Electrocardiography (ECG) was recorded continuously during scanning via a BrainAmp ExG MRI-compatible system (BrainProducts, Munich, Germany). The sampling rate was 5000 Hz. For each participant, bipolar Ag/AgCl electrodes were attached to the right and left side of the chest. Preprocessing of the ECG signal and RR interval analysis was performed similarly to Raz et al. (2012). Briefly, gradient artifacts were removed using FASTR algorithm (Niazy et al., 2005), implemented in FMRIB plug-in for EEGLAB (Delorme and Makeig, 2004). R peaks of ECG were detected using the FMRIB toolbox, and corrected for mis-detection (maximum correction rate over participants was 5.95%) and presence of ectopic beats. Finally, RR intervals were used to derive a beats-per minute HR index. Due to motion artifacts, 44 participants were included in the final HR analysis, for which a reliable R peak signal could be detected for all conditions.

### fMRI data acquisition and analysis

Brain scanning was performed on a 3T (GE, HDXt) MRI scanner with an 8-channel head coil. Functional imaging was acquired with gradient echo-planar imaging (EPI) sequence of T2^*^-weighted images (TR/TE/flip angle: 3000/35/90; FOV: 20 × 20 cm; matrix size: 96 × 96) in 39 axial slices (thickness: 3 mm; gap: 0 mm) covering the whole cerebrum. fMRI data analysis was performed with SPM5 (Wellcome Department of Imaging Neuroscience, London, UK). Preprocessing of the fMRI data included correction for head movements (subjects with movement above 2 mm were discarded) via realignment of all images to the mean image of the scan using rigid body transformation with six degrees of freedom, normalization of the images to Montreal Neurological Institute (MNI) space by co-registration to the EPI MNI template via affine transformation, and spatial smoothing of the data with a 6 mm FWHM. Finally, the first 6 images of each functional resting scan were rejected to allow for T2^*^ equilibration effects. Seed regions of interest (ROIs); the bilateral PCC and bilateral hippocampus, were defined anatomically and additionally masked to include gray matter only using the WFU Pick Atlas Tool (Maldjian et al., 2003, see also Stamatakis et al., 2010). To examine rsFC between seed ROIs and the whole brain, BOLD signal was filtered to low frequency fluctuations (0.01–0.08 Hz) using DPARSF toolbox (Chao-Gan and Yu-Feng, 2010). A mean time series across voxels in the seed ROIs was calculated for each participant using the MarsBaR software package (http://marsbar.sourceforge.net). GLM analyses were then performed between the ROI time series and the time series for each brain voxel. To reduce the effect of the physiological artifacts and nuisance variables, the whole-brain mean signal, six motion parameters, cerebrospinal fluid, and white matter signals were introduced as covariates in the design matrix (Chao-Gan and Yu-Feng, 2010).

First, random effect group analysis (RFX) was used to identify regions that altered connectivity to the seed ROIs when comparing rest conditions before and immediately after the stressful manipulation. In this RFX analysis a one sample *t*-test was applied to the images of contrast between the two rest sessions of all subjects (i.e., random subject effects with fixed condition effects). Next, these connectivity alterations were further explored in the third rest condition. Statistical maps for the PCC seed were corrected for multiple comparisons (FDR < 0.05) and the Statistical maps for the hippocampus seed were set at a threshold of *p* < 0.001, small volume corrected (with a cluster size of at least 20 voxels). The resulting brain areas were anatomically validated with the WFU Pick Atlas Tool. Beta weights were extracted and averaged across all voxels within each functional area that altered connectivity to the seed ROIs.

### Psychological assessment

Participants were asked to complete a self-report questionnaire to assess trait anxiety the State Trait Anxiety Inventory-Trait Version (STAI-T) (Spielberger, 1983).

### Statistical analyses

Neural, behavioral, and physiological measures were statistically analyzed using repeated-measures ANOVA (STATISTICA 7) to assess the effect of the experimental condition. The correlations between brain measures and individual cortisol response (AUCi) were assessed using Pearson's regression analysis followed by a hierarchical multiple regression (STATISTICA 7).

## Results

### Behavioral and physiological measures of stress induction

**Figure 2:**
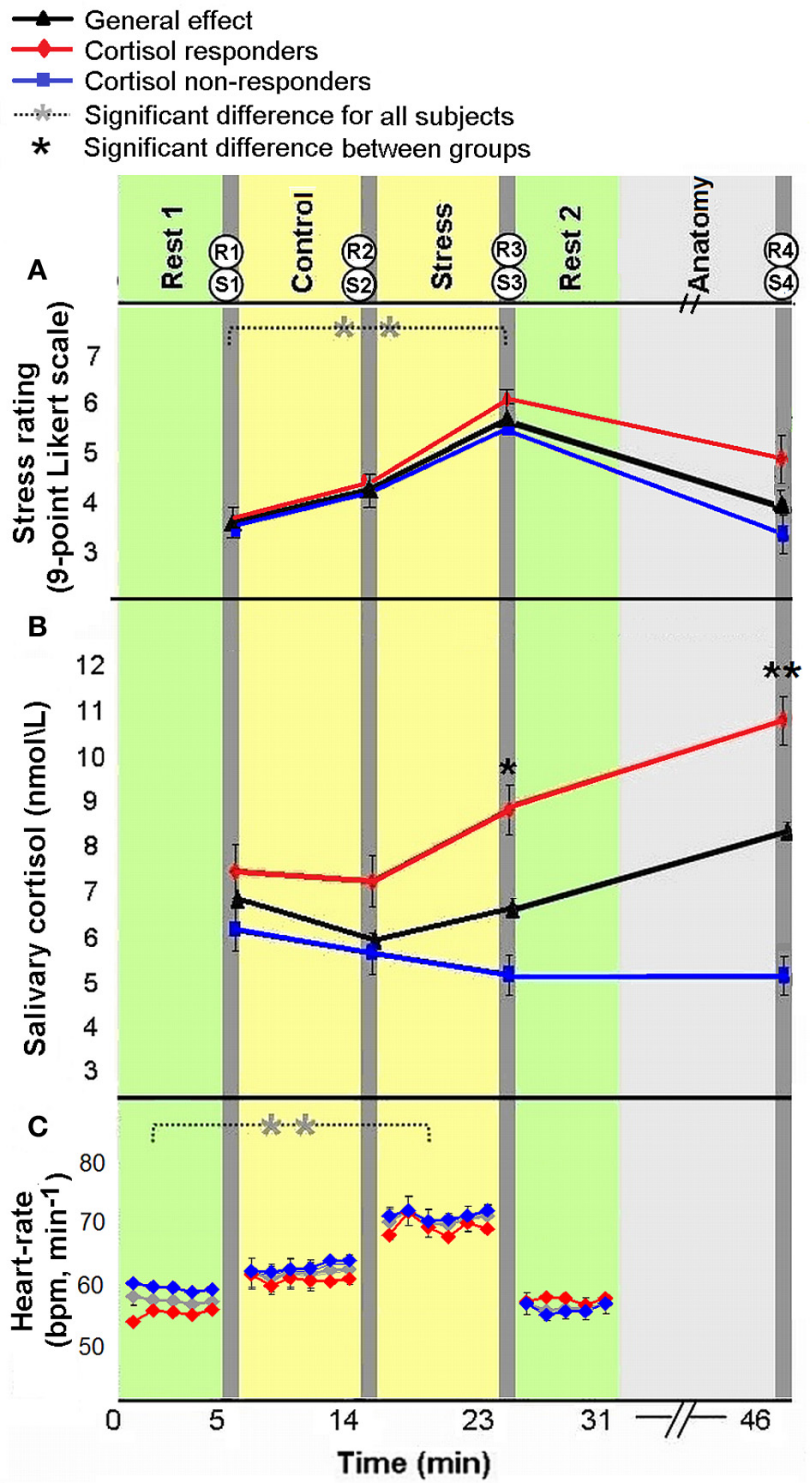
**Physiological and psychological response to stress.** Subjective ratings of stress **(A)**, average salivary-cortisol level **(B)**, and heart rate (HR, bpm) **(C)** presented in reference to the time course of the experiment. Time 0 indicates the start of experiment. The yellow columns represent control and stress tasks (6 min each), green columns represent “rest” conditions (fixation, open eyes, 5 min) and light gray column represents an anatomical scan (15 min). Between scans (dark gray columns), behavioral rating of stress [R(1–4)] and salivary-cortisol samples [S(1–4)] were taken. HR was continuously recorded. The error bars indicate standard error. ^*^p < 0.05, ^**^p < 0.001.

All measures of stress induction revealed a general effect of stress-elicitation at the group level. Specifically, a main effect of time was found for subjective ratings of stress [*F*_(3, 180)_ = 17.562, *p* < 0.001]; Fisher's least significant difference (LSD) *post-hoc* analyses revealed an increase in ratings in response to stress (R3) as compared to the two previous measures (R1 and R2, both *p*'s < 0.001), and a decline to baseline following the second rest period (R4, *p* < 0.001, Figure 2A). The means and standard deviation (in parenthesis) of R1, R2, R3, and R4 were 3.69 (1.82), 4.21 (1.96), 5.34 (1.9), and 4.08 (2.39), respectively. Notably, the four measures of subjective stress were within the normality range (values of Skewness and Kurtosis were within the range of ±2 standard errors). For salivary cortisol a marginally significant main effect of time was found [*F*_(3, 171)_ = 2.4579, *p* = 0.064; 4 participants were excluded from cortisol analysis due to insufficient saliva samples], according to *post-hoc* analysis, we found a marginally significant peak in cortisol level in the final sample (S4) as compared to post “rest 1” sample (S1, *p* = 0.057). In accordance with stress literature, two distinct cortisol groups emerged in response to stress: *responders*, who were defined by an increase of at least 1.5 nmol/L and a 15% rise from pre-stress levels (suggested earlier by Fehm-Wolfsdorf et al., 1993; Lupien et al., 1997; Schwabe et al., 2008) (38% of participants, *n* = 22); and *non-responders*, who showed no change or diminished cortisol level (62% of participants, *n* = 36). The analysis of cortisol levels at the 4 time periods by cortisol response groups revealed a main effect of group [*F*_(1, 56)_ = 9.64, *p* < 0.001], an effect of time of measurement [*F*_(3, 168)_ = 9.14, *p* < 0.001] and a significant interaction [*F*_(3, 168)_ = 34.81, *p* < 0.001]. For the *responders* group, Fisher's LSD *post-hoc* comparison revealed a significant increase in cortisol level 20 min following stress-induction (S4) relative to all previous levels (*p*'s < 0.001), and an increase post stress (S3) relatively to control (S2, *p* < 0.01). Whereas, a significant decrease in cortisol was found for *non-responders* at the two post stress measurements (S3, S4) relatively to post “rest 1” (S1) (both *p*'s < 0.005, Figure 2B, red and blue, respectively). A significant difference between groups was found for post stress sample (S3, *p* < 0.05) and final sample (S4, *p* < 0.001). The means and standard deviation (in parenthesis) of S1, S2, S3, and S4 for cortisol *responders* (in nmol\ lL) were 7.35 (2.13), 6.93 (2.42), 8.69 (4.10), and 10.81 (4.65), respectively. Means and standard deviation for cortisol *non-responders* were 6.46 (4.39), 5.83 (4.01), 5.08 (3.50), and 4.88 (3.02), respectively.

Finally, HR (beats per minute) analysis also revealed a main effect of time [*F*_(3, 129)_ = 38.88, *p* < 0.001; 44 participants with a reliable R peak signal were included in this analysis]; Fisher's LSD *post-hoc* analyses revealed an increase in HR in response to stress, as compared to pre-stress conditions (*p* < 0.001), and a decrease to initial levels during the second rest period (*p* < 0.001, Figure 2C). The means and standard deviation (in parenthesis) of the 4 HR measures (in bpm) were 57.97 (9.36), 65.25 (10.44), 69.39 (9.60), 58.99 (9.82), respectively. One of the 44 subjects included in HR analysis had no sufficient cortisol samples. Notably, as in the whole group (the 58 subjects for which we obtained reliable endocrine data), 17 of the 43 participants included in HR and cortisol analyses (39.53%) were cortisol *responders*. Importantly, in the repeated measures ANOVA analyses for HR and subjective stress measures, no significant interaction was found between group and time [*F*_(3, 123)_ = 1.09, *p* = 0.36; *F*_(3, 168)_ = 2.36, *p* = 0.074, respectively]. Additionally, *responders* and *non-responders* did not differ in measure of trait anxiety [*F*_(1, 56)_ = 0.158, *p* = 0.69].

### Early-stage resting state manifestations of stress

We first compared rsFC patterns with the bilateral PCC and hippocampus seed ROIs between “rest 1” and “rest 2” conditions (interspersed with the stressful arithmetic task), localizing immediate post-stress rsFC alterations. Peak voxels and corresponding *T*-values for all locations of the significant clusters are presented in Table 1. We next probed late-stage rsFC alterations, comparing “rest 2” (immediately following the stressor) and “rest 3” (2 h following the stressful task).

**Table 1:** **(A)** Peak voxels and corresponding *T*-values for regions that show altered rsFC with the PCC seed in the following contrasts; **(B)** Peak voxels and corresponding *T*-values for regions that show rsFC with the bilateral hippocampus in the following contrasts.

	**Hem**	**MNI coordinates**			***t*-value**
		***x***	***y***	***Z***	
**(A) “REST 2” > “REST 1” CONTRAST**				
Inferior parietal lobule	R	54	−63	39	3.89^**^
	L	−45	−69	42	4.03^**^
Thalamus	R	15	−12	18	5.09^**^
	L	−3	−12	12	4.58^**^
Caudate nucleus	R	9	0	15	5.08^**^
	L	−12	0	12	5.15^**^
Medial PFC	R	3	54	6	4.59^**^
**“REST 1” > “REST 2” CONTRAST**				
Posterior insula	R	36	−24	15	4.93^**^
	L	−42	−30	18	3.86^**^
Lingual gyrus	R	15	−51	−9	4.7^**^
	L	−15	−57	−9	4.58^**^
**(B) “REST 2” > “REST 1” CONTRAST**				
Amygdala	L	−21	−3	−21	4.85^*^
Middle temporal gyrus	R	42	−63	18	3.65^*^
**“REST 1” > “REST 2” CONTRAST**				
	None			

* p < 0.001, small volume corrected, with a cluster size of at least 20 voxels;

** p < 0.05 (FDR corrected).

**Figure 3:**
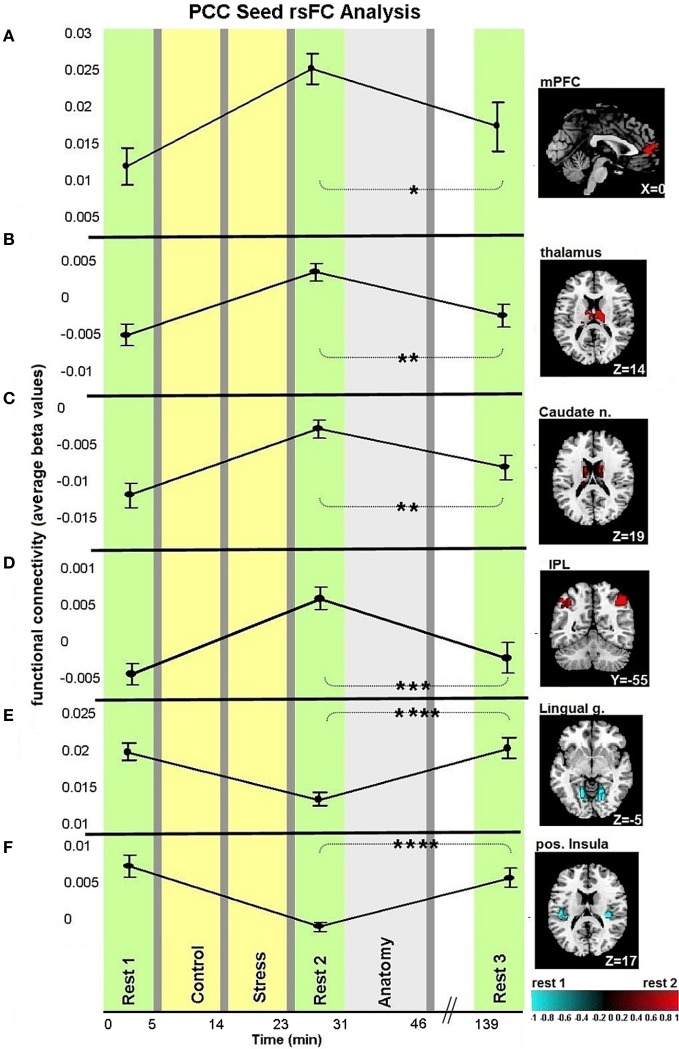
**Dynamics in rsFC to the PCC seed ROI in the three “rest” conditions.** Localized areas that changed connectivity to the PCC seed **(A–F)** when contrasting “rest 1” and “rest 2” are presented on the right hand side. rsFC at “rest 1,” “rest 2,” and “rest 3” are presented in reference to the timeline of the experiment. Statistic maps were corrected for multiple comparisons (FDR < 0.05) *T*-score scale is shown at the bottom. Error bars indicate standard error. ^*^p < 0.05, ^**^p < 0.01, ^***^p < 0.001, ^****^p < 0.0001. See Table 2 for specific *p*-values.

**Figure 4:**
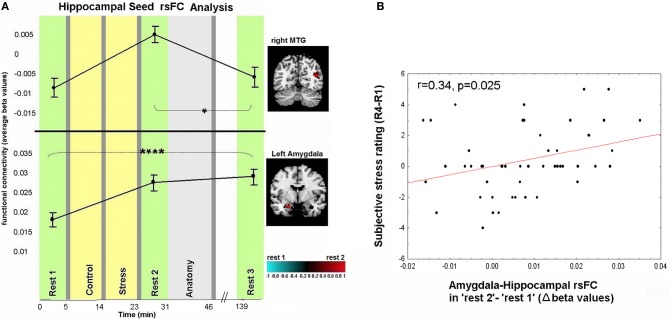
**Dynamics in rsFC to the hippocampus seed ROI and association between subjective stress and early-stage limbic connectivity. (A)** Localized areas that changed connectivity to the hippocampus seed when contrasting “rest 1” and “rest 2” are presented on the right hand side. rsFC at “rest 1,” “rest 2,” and “rest 3” are presented in reference to the timeline of the experiment. Statistic maps for the hippocampus seed were set at a threshold of *p* < 0.001, small volume corrected (with at least 20 voxels). *T*-score scale is shown at the bottom. Error bars indicate standard error. ^*^p < 0.05, ^****^p < 0.0001. See Table 2 for specific *p*-values. **(B)** Pearson correlation between subjective stress sensation (measured 20 min post-stress subtracting the initial rating post-“rest 1,” R4-R1), and early stress-induced change in amygdala-hippocampal rsFC as measured during “rest 2” relative to “rest 1.”

Figures 3, 4A demonstrate rsFC to the seed ROIs in the three rest conditions, in reference to the timeline of the experiment. Results from the first comparison indicate six brain areas that alter time course coupling to the PCC between “rest 1” and “rest 2” (Table 1A; Figures 3A–F, right hand side). The mPFC, thalamus, caudate nucleus and IPL increased their connectivity with the PCC following stress, whereas the posterior insula and lingual gyrus decreased their connectivity with the PCC; all regions showed bilateral effects. Two areas altered their rsFC with the hippocampus seed between “rest 1” and “rest 2,” the left amygdala and right middle temporal gyrus (MTG), both of which increased their connectivity with the hippocampus following stress (Table 1B; Figure 4A).

### Correlating subjective stress sensation to brain measures

An investigation of the relation between the psychological and neural measures of stress revealed that early-stage rsFC alterations in amygdala-hippocampal connectivity (contrasting “rest 2” and “rest 1”) is significantly correlated to changes in subjective stress (last sample, R4, vs. first sample, R1; *r* = 0.34, *p* = 0.025, Figure 4B). Notably, no significant correlations were obtained between the change in subjective stress and the difference in connectivity found between “rest 1” and “rest 2” over the MTG, mPFC, posterior insula, IPL, thalamus, lingual, and caudate (*p*-values = 0.19, 0.24, 0.09, 0.13, 0.25, 0.88, 0.22, respectively).

Considering the correlation between amygdala-hippocampal connectivity and the subjective stress report, we also investigated the relation between task performance (measured as number of mistakes) and this change in connectivity. We found no correlation between performance and limbic connectivity (*r* = −0.35, *p* = 0.120).

### Prolong alterations in rsFC to the seed ROIs

A repeated-measures ANOVA followed by Fisher's LSD *post-hoc* comparisons were used to detect differences between the third rest condition and the two previous ones. Regarding the PCC, when comparing “rest 3” to “rest 2,” all regions presented an opposing pattern of correlation compared to the pattern found between “rest 1” and “rest 2” conditions. When comparing “rest 3” to “rest 1,” no significant differences were found for PCC connectivity with all areas. Regarding the hippocampus seed, the right MTG presented the same opposing connectivity pattern when comparing “rest 3” to “rest 2.” Furthermore, MTG-hippocampal connectivity in “rest 3” decreased to initial “rest 1” levels. Nonetheless, as opposed to all other functionally connected areas presented in this study, only the amygdala-hippocampal connectivity showed a clear difference between “rest 3” and “rest 1” conditions, demonstrating a sustained increase (Table 2).

**Table 2:** **(A)** Fisher's LSD *post-hoc* comparisons between rsFC with the PCC seed ROI in different rest conditions; **(B)** Fisher's LSD *post-hoc* comparisons between rsFC with the hippocampus seed in different rest conditions.

**Conditions compared**	**“rest 3” to “rest 2” *p*-value**	**“rest 3” to “rest 1” *p*-value**
**(A)**		
Bilateral inferior parietal	0.0002^***^	0.359
lobule		
Bilateral thalamus	0.0019^**^	0.220
Bilateral caudate nucleus	0.0077^**^	0.122
medial PFC	0.036^*^	0.106
Bilateral posterior insula	0.27 × 10^−4^^****^	0.385
Bilateral lingual gyrus	0.35 × 10^−4^^****^	0.914
**(B)**		
Right middle temporal gyrus	0.032^*^	0.187
Left amygdala	0.61	2.71 × 10^−4^^****^

* p < 0.05;

** p < 0.01;

*** p < 0.001;

**** p < 0.0001.

### Amygdala-hippocampal sustained rsFC change and cortisol responsiveness

**Figure 5:**
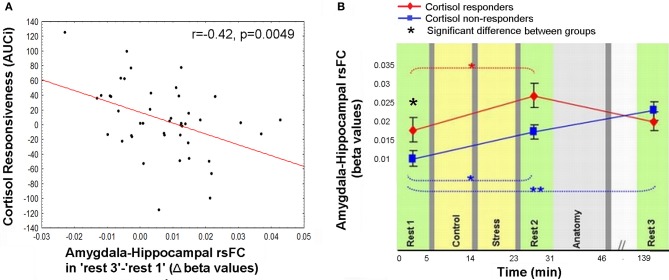
**Relation between cortisol response and prolong changes in amygdala-hippocampal rsFC. (A)** Negative correlation between individual cortisol response (AUCi value) and the sustained increase in amygdala-hippocampal connectivity as measured during “rest 3” relative to “rest 1”; **(B)** amygdala-hippocampal rsFC in the three rest conditions, calculated separately for cortisol *responders* (red) and *non-responders* (blue), and presented in reference to the timeline of the experiment. Between-groups differences were found in “rest 1” and “rest 2,” and within the *non-responders* group between “rest 1” and “rest 3.” The error bars indicate standard error. ^*^p < 0.05, ^**^p < 0.001.

The anomalous lingering rise in limbic rsFC led to the conjecture that the interactions of both the hippocampus and the amygdala with the hypothalamic-pituitary-adrenal (HPA) axis may contribute to this effect. This was explored by correlating the individual cortisol AUCi values with the degree of sustained change in amygdala-hippocampal connectivity in “rest 3” vs. “rest 1.” Three participants were excluded from this analysis as outliers due to beta values exceeding ±2.5 Std from group average. Taken together, the final analyses regarding the relation between limbic connectivity and cortisol included 45 subjects. The analysis revealed a significant negative correlation to cortisol responsiveness (*r* = −0.42, *p* = 0.0049, Figure 5A), suggesting that more cortisol secretion was associated with less limbic connectivity enhancement. No significant correlations were obtained between AUCi and the difference in connectivity between rest 1 and 3 over all other functional connections mentioned. We further investigated whether our behavioral measures of stress may have played a role in the sustained increase in limbic connectivity. A hierarchical regression was performed for predicting the change in limbic connectivity between “rest 1” and “rest 3.” The regression was computed in order to assess the added value of behavioral indices to the endocrine measure (AUCi). We tested the subjective stress rating (4 rating values) and trait anxiety (STAI-T score). To note, No correlation was found between predictors (all *p*'s > 0.23). At the first step AUCi was introduced, at the second step the group was introduced, at the third step the 4 rating values, STAI-T scores and at the fifth step the interaction between group and ratings (as 4 variables composed by the product between group and each of the 4 ratings). The effect of AUCi was significant when entered alone [in the first step, *F*_(1, 41)_ = 8.21, *p* = 0.007, *R* square = 0.167], however, when additional variables were added (since the second step) it's unique value in the explanation of the change in limbic connectivity in “rest 3” relative to “rest 1” was no longer significant.

To further specify the early and late-stage relations of amygdala-hippocampal rsFC and the cortisol response, connectivity was investigated separately for the two groups (i.e., cortisol *responders* and *non-responders*, Figure 5B). Results indicated a significant interaction between the strength of the amygdala-hippocampal rsFC in the different cortisol response groups and the timing of the rest condition [*F*_(2, 86)_ = 4.29, *p* = 0.016]. Fisher's LSD *post-hoc* analysis revealed that among *responders* limbic connectivity in “rest 3” decreased to “rest 1” levels, whereas among *non-responders* a sustained rise in amygdala-hippocampal connectivity was exhibited (*p* < 0.001). Additionally, *responders* had a significantly higher amygdala-hippocampal rsFC in “rest 1” compared to *non-responders* (*p* = 0.036). In “rest 3,” on the other hand there was no group difference in limbic connectivity.

## Discussion

### Early-stage alterations of rsFC following a stressful experience

We studied the trajectory of acute stress responsiveness in a group of 51 healthy males. As expected, our task induced stress, reflected in subjective reports and HR (Figure 2). Consistent with our hypothesis, both a network with a node in the PCC and a network with a node in the hippocampus showed immediate post-stress rsFC modulations (i.e., the difference between rsFC in “rest 2” vs. “rest 1”), though with different clusters of regions and dynamics. For the PCC seed, our results regarding early stage alterations indicate increased coupling with other major DMN nodes, including the mPFC and bilateral IPL, as well as with other areas (Figure 3). This generally extends prior work (Fransson, 2005; Jiao et al., 2011) and is consistent with suggestions of the importance of the PCC as a DMN node that directly interacts with all other network nodes (Fransson and Marrelec, 2008). Increased DMN connectivity in successive rest following a cognitive task is supported by previous studies (Waites et al., 2005; Pyka et al., 2009). Here we show the enhancement of DMN connectivity in the immediate aftermath of a socially stressful experience. Increased PCC coupling with other DMN nodes may reflect engagement of neural processes supporting self-referential mental processes and immediate reflections on the preceding stressful experience, possibly with regards to previous experiences (Fransson, 2005). Notably, modifications of coupling with the PCC occurred also in areas not included in the DMN, such as the caudate nucleus and posterior insula. These brain areas have been previously shown to have a strong presence in a PCC related network (Greicius et al., 2007; Uddin et al., 2009; Grigg and Grady, 2010). Specifically, diminished connectivity of the PCC with the posterior insula in the early aftermath of stress might reflect on the relocation of brain processing resources due to the enhanced cognitive and emotional demands related to task performance under a stressful and socially critical atmosphere.

Additionally we found increased early-stage rsFC of the hippocampus with the right MTG and left amygdala (Figure 4A). The hippocampal contribution to DMN has been attributed to its involvement in episodic memory (Greicius et al., 2004). The MTG has also been linked to the core DMN (Buckner et al., 2008) and thus its connectivity modification with the hippocampus might relate to the same mental reflection processes described above. The amygdala, on the other hand, is less commonly regarded as part of the DMN. In fact, amygdala-hippocampal pairing is considered a major limbic pathway for generation and regulation of emotional reaction in response to stressful stimuli (LeDoux, 2000). Support of this is shown by the correlation found between the reported subjective stress and early-stage rise in limbic connectivity (Figure 4B). Furthermore, enhancement in amygdala-hippocampal connectivity was suggested to be required for both emotional memory encoding and consolidation (Richter-Levin and Akirav, 2000; Roozendaal et al., 2003; Dolcos et al., 2004; Phelps, 2004; Smith et al., 2006). Taken together, we believe that the demonstrated early increase in intra-limbic connectivity may be related to the major role these two regions play in the memory processes of stressful events, encouraging future studies to address this issue.

### Late-stage alterations of rsFC following a stressful experience

Our study design enabled the identification of recovery patterns of rsFC with the PCC and hippocampus, as measured 2 h following the period of induced stress, considered here to be late stage modulation. To our knowledge, this time window of rsFC recovery following stress has not been previously examined in humans. Notably, both increased and decreased connections to the PCC in the early aftermath of stress returned to their initial levels once the last sampling point was reached (Figure 3). The post-task recovery nature of the DMN has been recently demonstrated by Barnes and colleagues, who also found that a more demanding task was followed by a slower recovery pattern, as compared to an easier task (Barnes et al., 2009). This recovery occurred on a scale of minutes, yet stress was shown to induce alterations in rsFC even an hour following task performance (Veer et al., 2011). Uniquely, we demonstrate the late comprehensive recovery nature of rsFC following a documented stressful arousing experience. Our observation emphasizes the notion that the brain has the capacity to recover and restore homoeostasis over time. However, contrary to our expectations, the revealed dynamics of recovery, related to co-activation with the PCC, was neither related to subjective stress report nor to cortisol response.

On the other hand, the increase in the rsFC between the amygdala and hippocampus was sustained even 2 h after stress induction. Moreover, the lingering increase in connectivity between these two major limbic nodes was inversely related to the level of secreted cortisol in response to the stressful challenge (AUCi, Figure 5A). The hierarchical regression we performed pointed to the individual AUCi value as the only contributing factor to the sustained limbic connectivity. This result is in line with the attenuated positive connectivity previously found between the amygdala and the hippocampus following hydrocortisone intake (Henckens et al., 2012). In accordance with the notion that people may be grouped as cortisol *responders* and *non-responders* to induced stress, we unraveled that only the *responders* exhibited a recovery pattern of amygdala-hippocampal connectivity 2 h post-stress (Figure 5B). In other words, the persistently elevated limbic rsFC seemed to be selective to the group who did not exhibit increased cortisol secretion in response to acute stress. Since no difference was found between groups in repeated measure analysis of subjective stress rating and HR, we can assert, in accordance with previous studies that the cortisol effect is separate from autonomic and behavioral measures of arousal (Schwabe et al., 2008).

What might such interpersonal variability in cortisol response represent? Reciprocal interactions exist between both the amygdala and the hippocampus and the HPA-axis, which stimulates these elements and is regulated by them (Tsigos and Chrousos, 2002). The limbic network, high in glucocorticoid receptors, influences the activation of the HPA-axis, and these afferent pathways are exposed to the concentrations of the axis end-effector; cortisol (De Kloet et al., 2005). Therefore, causal factors contributing to our results may derive from both the variations in limbic connectivity and the degree of negative feedback exerted by cortisol secretion. The significantly higher limbic connectivity in the *responders* group found before the actual stress induction (in “rest 1,” Figure 5B), might have a pivotal contribution to the tendency to increase cortisol secretion. From a reciprocal perspective, we also presume that cortisol may have played an essential role in the regulation and balance of limbic interregional connectivity in the *responders* group when the stressor has gone. Whereas the lack of increase in cortisol among the *non-responders*, may have led to the demonstrated delayed rise in limbic rsFC and possibly to slower neural recovery.

The correlation found between early-stage post-stress limbic connectivity and the subjective report of stress sensation (Figure 4B) led us to presume that a reduction in prolonged limbic rsFC may be an indication of a reduced sensation of stress. This assumption is in line with the study of Het and colleagues, which presented an association between cortisol and attenuated negative affect (measured by Positive and Negative Affect Schedule) in response to acute stress [TSST; (Het et al., 2012)]. However, subjective stress was not rated following “rest 3.” Thus, future studies may test our proposal on the dependence between cortisol stress-induced secretion and the dynamics of neural recovery from stress, with regard to long-term psychological outcomes following stressful encounters.

Studies have shown that cortisol secretion following an arousing stimuli increases consolidation and attenuates long-term recall of emotional context, as reviewed by (Wolf, 2009); in addition, this effect is presumed to depend on the interaction between the amygdala and hippocampus (Roozendaal et al., 2003). Clinical trials suggest that post-exposure treatment with mild doses of cortisol might be beneficial in patients suffering from psychiatric conditions in which aversive memories are at the core of the problem [e.g., social phobia (Soravia et al., 2006) or PTSD (Aerni et al., 2004)]. Our results of inverse relations between post-stress sustained limbic connectivity and the cortisol response to stress might therefore add a new vantage point for future studies of the effects of stress on memory.

To note, our study was conducted on military soldiers in training, prior to operational employment. At the time of the experiment subjects were positioned in the same unit, thereby presenting a high homogeneity of life events over the months preceding our study. Nonetheless, we encourage future validation of our results on civilian populations as well. Additionally, an intriguing issue for further exploration is the responsiveness of our subjects, as well as their inter-individual differences following operational employment; a period that tends to include numerous life-threatening and stressful events.

In summary, our multiple time-point study demonstrates both early and late effects of a stressful challenging task on interregional rsFC. Our observations have important implications for the broader understanding of the impact of acute stress, and thus may be of substantial value in the search for a neuro-endocrine individual profile of stress responsiveness and related psychopathologies.

### Conflict of interest statement

The authors declare that the research was conducted in the absence of any commercial or financial relationships that could be construed as a potential conflict of interest.
